# Long-term survival after resection of pancreatic ductal adenocarcinoma with para-aortic lymph node metastasis: case report

**DOI:** 10.1186/1477-7819-11-195

**Published:** 2013-08-14

**Authors:** Toshihiko Masui, Toyonari Kubota, Keiko Aoki, Yasutaka Nakanishi, Takumi Miyamoto, Junko Nagata, Koshiro Morino, Atsushi Fukugaki, Michio Takamura, Shinichi Sugimoto, Hideyuki Onuma, Atsuo Tokuka

**Affiliations:** 1 Department of Surgery, Kyoto University, Kyoto, Japan; 2 Department of Surgery, Shimane Prefectural Central Hospital, Izumo, Japan; 3 Department of Pathology, Shimane Prefectural Central Hospital, Izumo, Japan

**Keywords:** Pancreatic cancer, Para-aortic lymph node metastasis

## Abstract

Pancreatic cancer patients with para-aortic lymph node metastasis have a poor prognosis and patients living longer than 3 years are rare. We had a patient with pancreatic cancer who survived for more than 10 years after removal of the para-aortic lymph node metastasis. A 57-year-old woman was diagnosed with pancreatic head cancer and underwent a pancreaticoduodenectomy with subtotal gastric resection following Whipple reconstruction in 2000. Para-aortic lymph node metastasis was detected during the operation by intraoperative pathological diagnosis and an extended lymphadenectomy was performed with vascular skeletonization of the celiac and superior mesenteric arteries. In 2004, a low-density area was detected around the superior mesenteric artery (SMA) 5 cm from its root and she was treated with gemcitabine, and the area was undetectable after 3 years of treatment. In 2010, computed tomography showed a low-density area around the same lesion with an increased carcinoembryonic antigen level. After 4 months of gemcitabine treatment, we resected the tumor *en bloc* with the associated superior mesenteric vein and perineural tissue. Histopathological examination of the resected specimen revealed a well-differentiated tubular adenocarcinoma that closely resembled the original primary pancreatic cancer, indicating perineural recurrence 10 years after the initial resection. She had no recurrence around the SMA for more than one year. Although a meta-analysis has not proved the efficacy of preventive radical dissection, this case indicates that a patient with well-differentiated, chemotherapy-responsive pancreatic cancer with para-aortic lymph node metastasis could have a long survival time through extended dissection of the lymph nodes.

## Background

Pancreatic cancer is among the leading causes of cancer death in Japan and worldwide. Even with a curative resection, the overall 5-year survival rate is reported to be only 6.8% to 25% and the median survival time of resected pancreatic cancer patients is 8 to 12 months [1-6]. For pancreatic cancer, patients with metastasis to the para-aortic lymph node have apparently shorter survival times compared to patients without metastasis, implicating the systemic spread of the tumors. Several previous reports have indicated a median survival time (MST) of 5 to 12 months after resection in metastatic para-aortic lymph node patients (Table 1). Most of these patients survived no more than 3 years. We report the case of a patient who survived for more than 10 years after pancreaticoduodenectomy for pancreatic cancer with para-aortic lymph node metastasis. Moreover, during follow-up a tumoral recurrence occurred 10 years later, which was successfully resected.

**Table 1:** **Median survival time of patients affected by pancreatic ductal adenocarcinoma with para**-**aortic lymph node metastasis**

**Author**	**Year**	**Number of patients**	**Percentage with PALN metastasis**	**Three-year survival with PALN metastasis**	**Median survival time (months) with PALN metastasis**
Murakami *et al*. [7]	2009	103	17%	0%	12.4
Doi *et al*. [8]	2007	133	14%	0%	5.1
Shimada *et al*. [9]	2006	133	20%	0%	13
Sakai *et al*. [10]	2005	178	19%	3%	NR
Yoshida *et al*. [11]	2004	34	26%	0%	NR
Kayahara *et al*. [12]	1999	99	18%	11%	NR

*NR* not reported; *PALN* para-aortic lymph node.

## Case presentation

**Figure 1:**
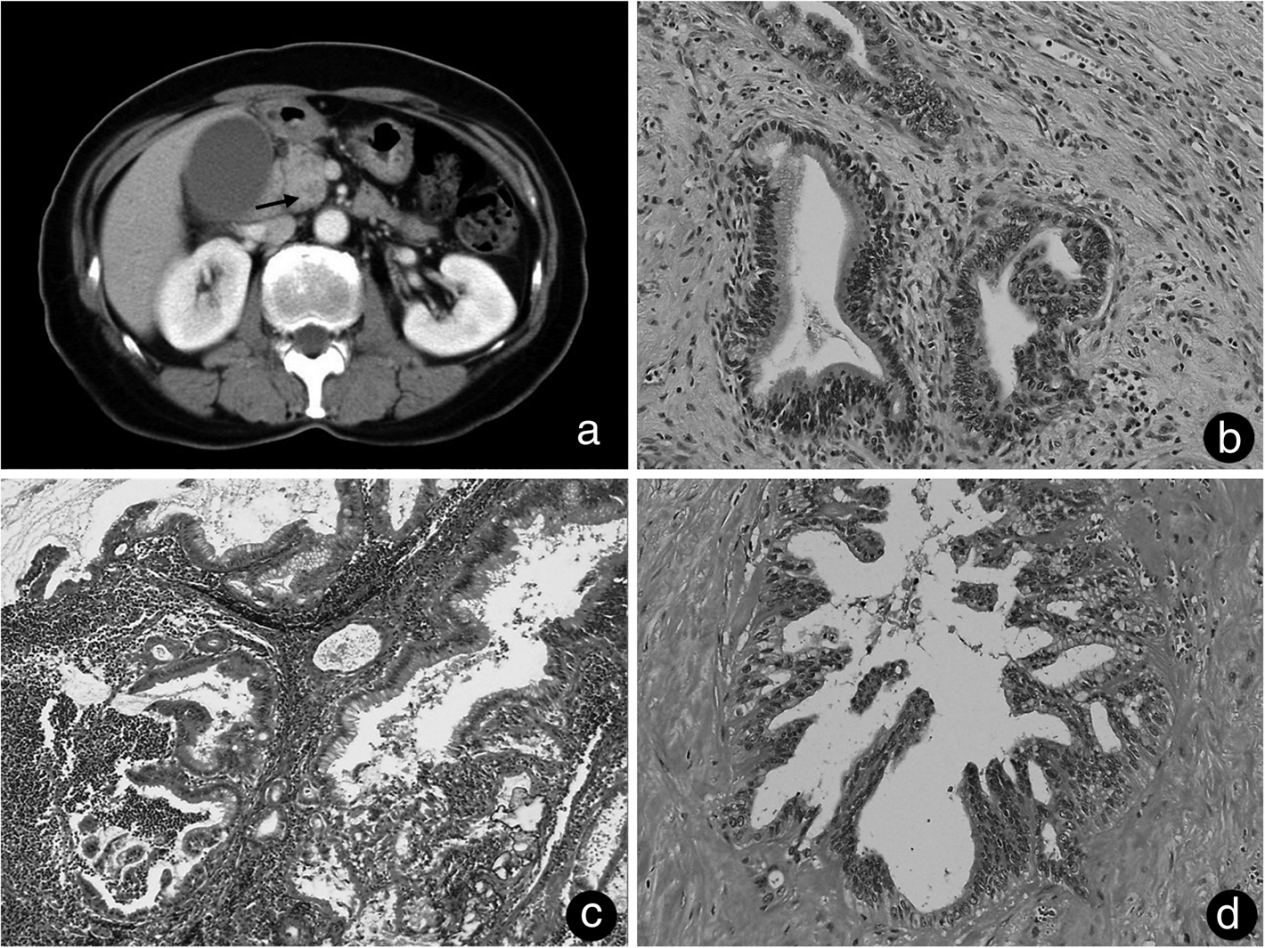
**Primitive tumor CT imaging and histopathological findings. (a)** Computed tomography on admission showed a 22 mm × 25 mm low-density mass around the uncus of the pancreas (arrow). **(b)** Microscope analysis showed a well-differentiated tubular adenocarcinoma surrounded by an extracellular matrix (hematoxylin and eosin, ×40). **(c)** The para-aortic lymph node had a well-differentiated tubular adenocarcinoma. **(d)** Resected perineural tissue with a histologic structure similar to the primitive tumor with hyalinization.

A 57-year-old woman with a history of jaundice was admitted to our hospital in October 2000, and an abdominal computed tomography (CT) scan showed a low-density mass of the pancreatic head (Figure 1a). No lymph node metastasis was detected by preoperative CT or Ultrasound Sonography (US).

No specific abdominal tumor was palpable by physical examination. No superficial lymph nodes were palpable. There was only a slight tenderness on the upper abdomen. Laboratory findings indicated slightly high levels of total bilirubin and amylase. The levels of sialyl Lewis A (CA19-9) and carcinoembryonic antigen (CEA) were normal. In December 2000, we performed a pancreaticoduodenectomy and a subtotal gastric resection. No ascites or peritoneal neoplastic dissemination were found. The tumor was palpable at the pancreatic head. There was no serosal invasion, but a retroperitoneal invasion was observed. We performed an extended lymphadenectomy and exeresis of the para-caval and para-aortic lymph nodes, because of the intraoperative pathological diagnosis of metastasis to the para-aortic lymph nodes (Figure 1c). We also performed a neural dissection around the root of the SMA.

The size of the tumor was 2.8 × 2.0 × 2.0 cm. Histopathological analysis revealed the tumor to be a well-differentiated tubular adenocarcinoma (Figure 1b). The tumor and the lymph node metastasis stained positive for cytokeratin AE3/AE4, which implies an epithelial origin (data not shown). The tumor histologically showed vascular, lymphatic and neural invasion and no cancerous region was found at around the resection edge. Metastasis was observed in lymph nodes 12b, 16b1 and 16a2 (2/3, 2/2 and 3/4 respectively) (Figure 1c). The final International Union Against Cancer tumor-node-metastasis classification was T3N1M1 (LYM) stage 4.

**Figure 2:**
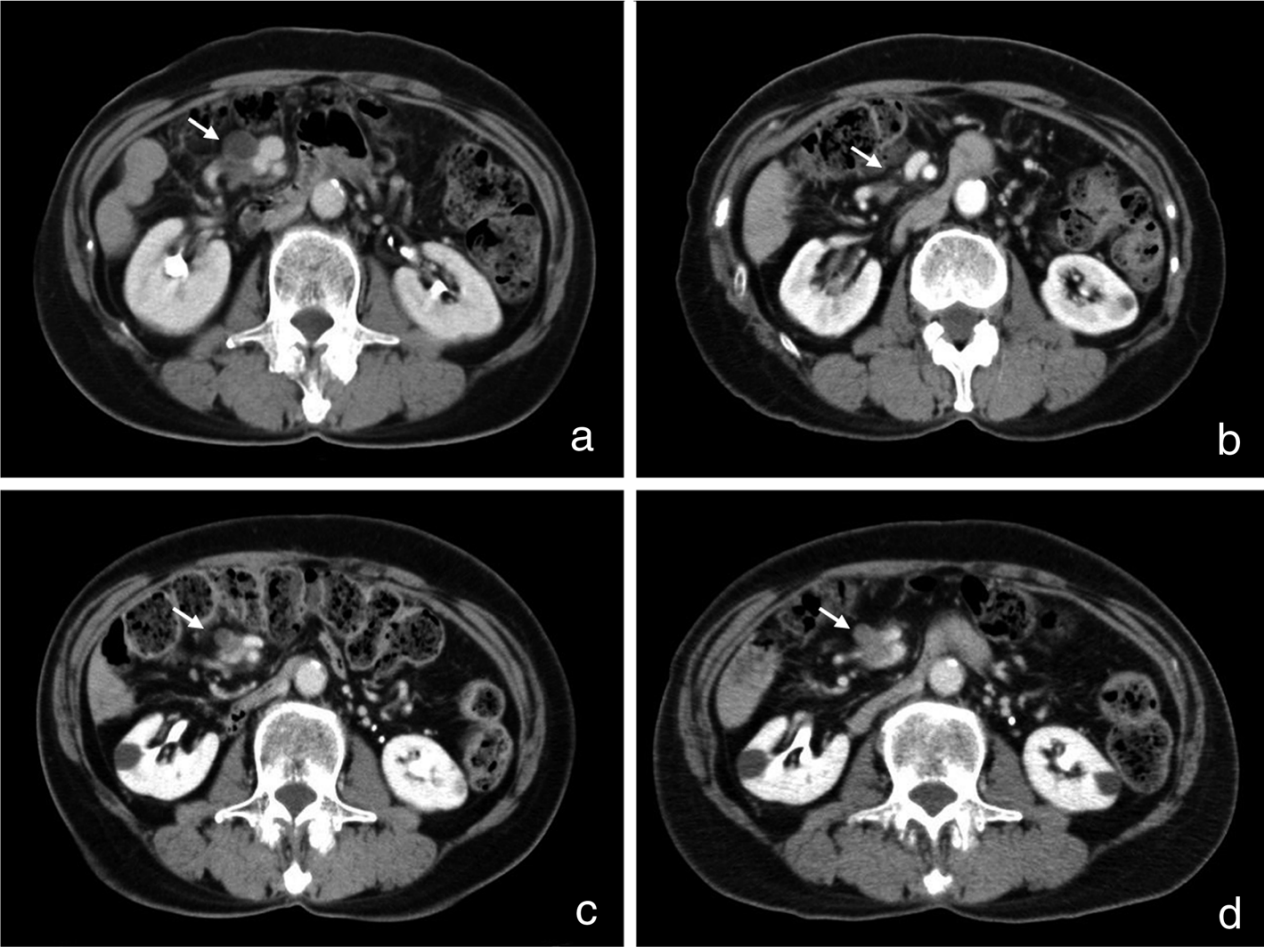
**Low-density mass around SMA with chemotherapy. (a)** CT examination revealed a low-density area around the SMA in 2004. **(b)** The tumor disappeared gradually over 3 years (to 2007). **(c)** Routine CT examination showed a low-density area around the SMA on August 2010. **(d)** The tumor was within the stable disease range after 4 months of gemcitabine treatment. Arrows show the low-density area around the SMA, which appeared after the first operation.

**Figure 3:**
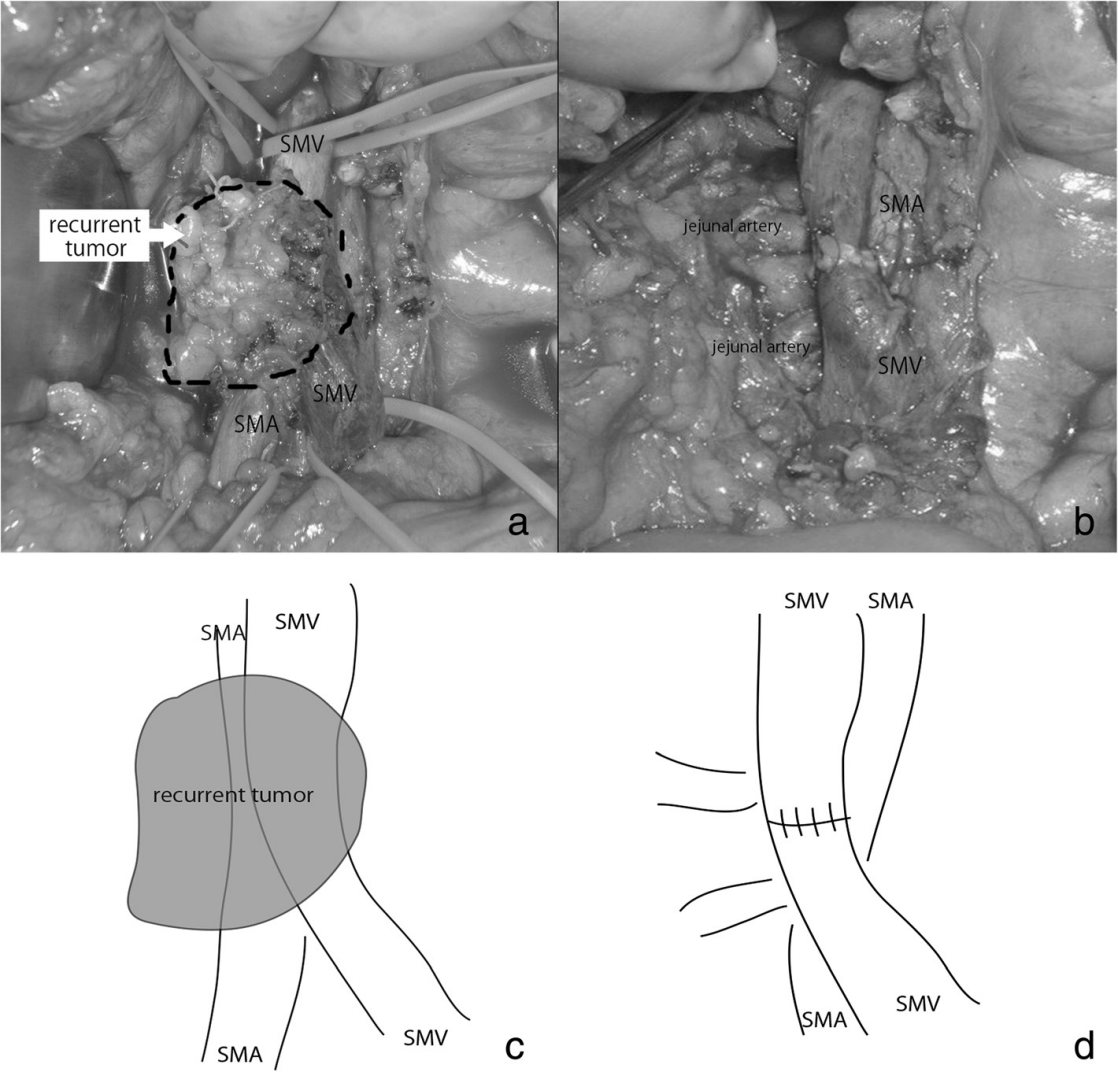
**Intraoperative findings. (a)** The tumor was tightly connected to the perineural tissue around the SMA with invasion of the SMV. **(b)** The tumor was resected with the perineural tissue and the SMV. **(c,d)** illustration of **(a)** and **(b)**. SMA, superior mesenteric artery; SMV, superior mesenteric vein.

The postoperative course was uneventful and the patient was discharged without postoperative chemotherapy because there was no standard adjuvant therapy at that time. In 2004, CT examination revealed a low-density area around the superior mesenteric artery (SMA) 5 cm from its root, suggesting lymph node metastasis (Figure 2a). She was treated with gemcitabine for 3 years. The tumor disappeared gradually (Figure 2b). From 2007 to 2010, there was no sign of recurrence even without chemotherapy. In August 2010, a routine CT examination displayed a low-density area around the SMA (Figure 2c) with a CEA level above the reference range (13 U/ml). We treated her with gemcitabine for 4 months, and no other metastases were found. The tumor itself was within the stable disease category (Figure 2d). In December 2010, we resected the tumor around the SMA. There was no peritoneal dissemination or liver metastasis. The tumor was tightly connected to the perineural tissue around the SMA and had invaded the superior mesenteric vein (SMV) (Figure 3a). However, it had not infiltrated the outer membrane of the SMA. The tumor was resected *en bloc* with the perineural tissue and the SMV, which was reconstructed by end-to-end anastomosis (Figure 3b). Histopathological examination of the resected specimen revealed a well-differentiated tubular adenocarcinoma very similar to the primitive pancreatic cancer (Figure 1d). Hyalinized tissue surrounded the tumor, suggesting tumor regression through chemotherapy. During the operation the tumor looked like a lymph node metastasis according to its CT localization. Histopathological examination showed a pancreatic tumoral origin from the perineural tissue of the SMA with invasion of the inner wall of the SMV. The postoperative course was uneventful and the patient recovered. An additional adjuvant treatment with gemcitabine was given. The patient was recurrence free for more than 1 year.

## Discussion

In patients with adenocarcinoma of the head of the pancreas, one of the factors predicting long-term survival is the absence of metastatic lymph nodes [13,14]. Therefore, to increase survival, extended lymphadenectomies have been performed since the 1990s. However, this extended procedure was not found to be beneficial for overall survival and seemed to be associated with increased morbidity compared with the standard procedure [15].

Para-aortic lymph node metastasis has been observed in around 14% to 26% of patients who underwent pancreaticoduodenectomy [7-12]. Thus far, there have been six retrospective surveys on the survival of patients with pancreatic ductal adenocarcinoma with para-aortic lymph node metastasis, and no patient survived longer than 5 years (Table 1). However, our patient survived for more than 10 years, although the tumor recurred in the perineural tissue of the SMA. Several factors for long survival times for pancreatic cancer patients have been reported, including a low pre CA19-9 level, the negativity of the dissected margin of the tumor [16], tumor differentiation [2,6,17] and the number of lymph node metastases [14]. Massucco *et al*. claimed that not only the number but also the localization of the metastases determines the prognosis [18]. In our case, we assume that the long survival time is due to the characteristics of the tumor and the extent of the operation: (1) the original tumor was a well-differentiated adenocarcinoma, (2) it showed good response to chemotherapy and (3) an extended lymphadenectomy was performed, which achived a negative margin status.

Egawa *et al*. reported to the National Pancreatic Cancer Registry that patients living longer than 5 years after curative resection are those who have well-differentiated primitive tumors, such as a papillary carcinoma or a well-differentiated adenocarcinoma, with good chemosensitivity [2,17]. Our patient had a well-differentiated carcinoma and was treated with gemcitabine for 3 years. The perineural recurrence around the SMA almost completely vanished with this treatment. The long survival time is apparently due to the good response of the tumor to the gemcitabine treatment. However, after a 2-year interval, the tumor recurred again and showed no progression or regression on further treatment with gemcitabine. Therefore, surgical resection was deemed necessary. Recently Marechal *et al*. reported that high activities of gemcitabine transport and metabolism proteins are important for long survival times after gemcitabine adjuvant therapy [19], suggesting that a tumor that responds well to gemcitabine treatment is genetically different from a non-responder. Genetic alterations that affect a tumor’s response to gemcitabine treatment should be analyzed when selecting patients for extended lymphadenectomy.

Also, the extended lymphadenectomy seemed to be important for our patient. Indeed, we did not find any recurrent tumor around the aorta but in the perineural tissue around the SMA. Although randomized trials have concluded that the addition of extended lymphadenectomy and retroperitoneal soft tissue clearance does not significantly improve overall survival [15], surgical resection has provided the only chance for long-term survival in pancreatic cancer patients, and exeresis of the metastatic lymph node may be worthwhile for patients with chemotherapy-responsive and well-differentiated pancreatic cancer defined by ultrasound-guided fine needle aspiration cytology.

Recently, Kato *et al*. reported that initially unresectable pancreatic cancer patients with a long-term favorable response to chemotherapy have long survival times after adjuvant surgery [20]. They found 50% of these patients survived for more than 5 years. Interestingly, their analysis showed that distant metastasis, such as peritoneal dissemination and liver metastasis rather than local invasion, results in poor prognosis. But they found only one case of metastasis in the para-aortic lymph nodes. In our case, the tumor recurring around the SMA had a good response to gemcitabine and was successfully resected with at least 1 year of disease-free survival. Therefore, neoadjuvant chemotherapy or adjuvant surgery could be an effective choice for patients with local recurrence even for pancreatic cancer.

## Conclusions

In conclusion, we observed a case of pancreatic cancer complicated by a para-aortic lymph node metastasis. After an extended lymphadenectomy, the patient survived for more than 10 years. According to our experience, for a local tumoral recurrence treated by chemotherapy, a further improvement in survival can be achieved by carefully selecting patients to undergo adjuvant surgical treatment.

## Consent

Written informed consent was obtained from the patient for the publication of this report and any accompanying images.

## Abbreviations

CEA: Carcinoembryonic antigen; CT: Computed tomography; MST: Median survival time; SMA: Superior mesenteric artery; SMV: Superior mesenteric vein.

## Competing interests

Toshihiko Masui and the other co-authors have no conflicts of interest.

## Authors’ contributions

TM, TK, KA, YN, TM, JN, KN, AF, MT, SS, AM participated in the treatment of the patient. HO carried out the pathological diagnosis. All authors read and approved the final manuscript.
